# A direct association between amber and dinosaur remains provides paleoecological insights

**DOI:** 10.1038/s41598-019-54400-x

**Published:** 2019-11-29

**Authors:** Ryan C. McKellar, Emma Jones, Michael S. Engel, Ralf Tappert, Alexander P. Wolfe, Karlis Muehlenbachs, Pierre Cockx, Eva B. Koppelhus, Philip J. Currie

**Affiliations:** 1 Department of Palaeontology, Royal Saskatchewan Museum, 2340 Albert Street, Regina, Saskatchewan S4P 2V7 Canada; 2 Division of Entomology, Natural History Museum, and Department of Ecology & Evolutionary Biology, 1501 Crestline Drive – Suite 140, University of Kansas, Lawrence, Kansas 66045 USA; 3 Biology Department, University of Regina, Regina, Saskatchewan S4S 0A2 Canada; 4 Department of Earth and Atmospheric Sciences, University of Alberta, Edmonton, Alberta T6G 2E3 Canada; 5 Geology Department, Lakehead University, Thunder Bay, Ontario P7B 5E1 Canada; 6 Department of Biological Sciences, University of Alberta, Edmonton, Alberta T6G 2E9 Canada

**Keywords:** Palaeontology, Palaeoecology

## Abstract

Hadrosaurian dinosaurs were abundant in the Late Cretaceous of North America, but their habitats remain poorly understood. Cretaceous amber is also relatively abundant, yet it is seldom found in direct stratigraphic association with dinosaur remains. Here we describe an unusually large amber specimen attached to a *Prosaurolophus* jaw, which reveals details of the contemporaneous paleoforest and entomofauna. Fourier-transform Infrared spectroscopy and stable isotope composition (H and C) suggest the amber formed from resins exuded by cupressaceous conifers occupying a coastal plain. An aphid within the amber belongs to Cretamyzidae, a Cretaceous family suggested to bark-feed on conifers. Distinct tooth row impressions on the amber match the hadrosaur’s alveolar bone ridges, providing some insight into the taphonomic processes that brought these remains together.

## Introduction

Although dinosaur and resin fossils are abundant in the Late Cretaceous of western Canada, they are rarely associated, because conditions for their preservation differ. Thus, bone and amber records represent largely independent, albeit complementary, sources of information on different aspects of ancient ecosystems^1^. Despite the growing number of amber localities known in western Canada, insect-bearing amber in direct association with a dinosaur dig has only been reported once^1–4^. No finds detail the circumstances by which amber and dinosaurs come together in bonebeds, and only a single study examines what one reveals about the paleoecology of the other^1^. Excavation in 2010 within Dinosaur Provincial Park, Alberta^5^ recovered an isolated left hadrosaur dentary, UALVP 53367, in the Campanian uppermost Dinosaur Park Formation (~75 Ma). This site is immediately beneath the Lethbridge Coal Zone, within a series of muddy overbank facies that have been interpreted as products of an alluvial-coastal plain undergoing transgression^6^. Based on morphology, the dentary was identified as belonging to the hadrosaur *Prosaurolophus maximus* Brown, which is consistent with the known stratigraphic ranges of hadrosaurs within the region. Preparation revealed an exceptionally large mass of amber (~300 g), tightly adpressed to the alveolar bone, near the anterior margin of the lingual surface. This is one of the largest amber specimens documented from the Late Cretaceous of western Canada. Moreover, it provides both an insect inclusion and details on the habitat and taphonomy of the hadrosaur. The amber’s chemotaxonomic and stable isotopic composition provide insights into forest ecology and attendant climatic conditions. Observations of the hadrosaur bone and amber mass tie these details to the taphonomic events that created the association.

## Results

### Amber and bone association

**Figure 1:**
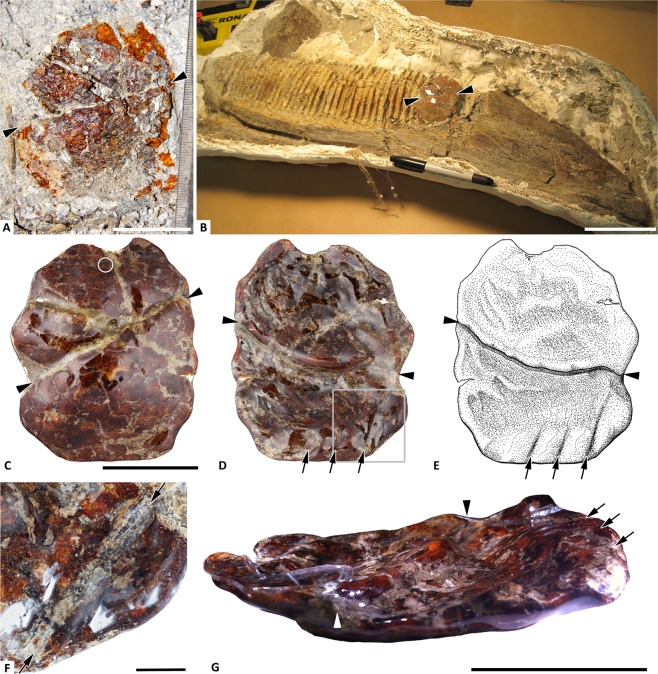
UALVP 53367 surface features. **(A)** Initial exposure of friable amber during fossil preparation, lingual surface. **(B)** Amber with epoxy support prior to removal from dentary. **(C)** Lingual surface, circle indicates aphid, arrowheads fault line. **(D)** Labial surface, arrows mark tooth row impressions. **(E)** Illustration corresponding to D. **(F)** Detail of deep alveolar ridge impression between arrows (on lower right of D and E). **(G)** Oblique view of labial surface highlighting fault and depressions, with margin contacting dentary to right. Scale bars 3 cm **(A, C–E, G)**, 10 cm **(B)**, 5 mm (**F**).

The discoidal amber piece is more than 7 cm wide, but only 8 mm in thickness. The half closest to the dentary was pushed underneath the other half, leaving a large ridge on the concave surface. This offset appears to be the product of compaction during burial and after resin solidification, because it is linear and does not distort flow lines within the amber. The ventral margin of the amber piece made direct contact with the dentary and it records three faint and one deep impression from tooth rows in the alveolar bone exposed after the dentary had lost its teeth (Fig. 1). The amber piece was stabilized with epoxy before it was separated from the dentary, but its ventrally directed margin was in contact with the bone, with less than one millimeter of sediment was trapped between the amber and the dental battery in the deepest recesses of the alveolar sockets. Specimen preparation has reduced some of the topography within the amber piece, but a series of three blade-shaped impressions are still visible along the ventral margin, with spacing, orientation, and shapes that directly correspond to the underlying bone ridges.

**Figure 2:**
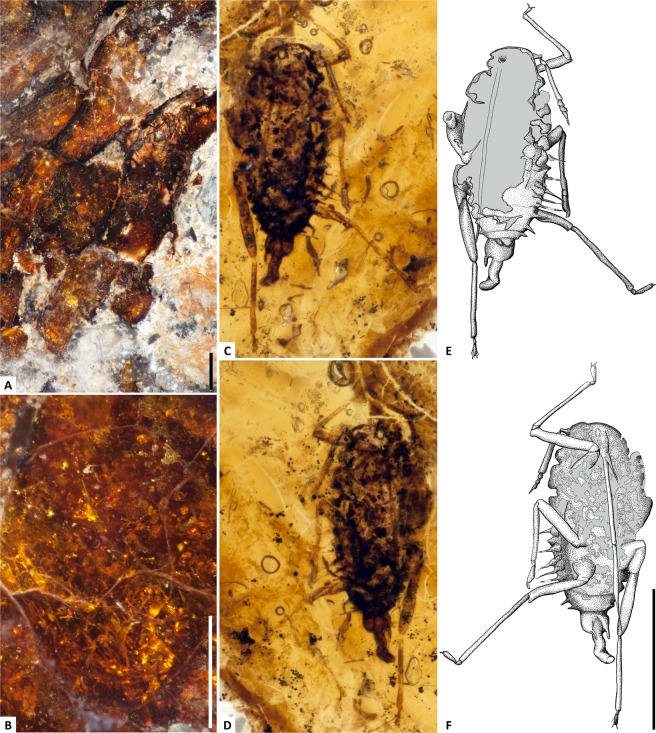
UALVP 53367 inclusions. **(A**) Sediment and carbon film between amber and dentary. **(B)** Fungal hyphae or flow lines amidst fractures. (**C–F)** Cretamyzidae dorsal and ventral photomicrographs with corresponding habitus diagrams; grey indicates missing material. Scale bars 1 mm **(A,B)**, 0.5 mm **(C–F)**.

**Figure 3:**
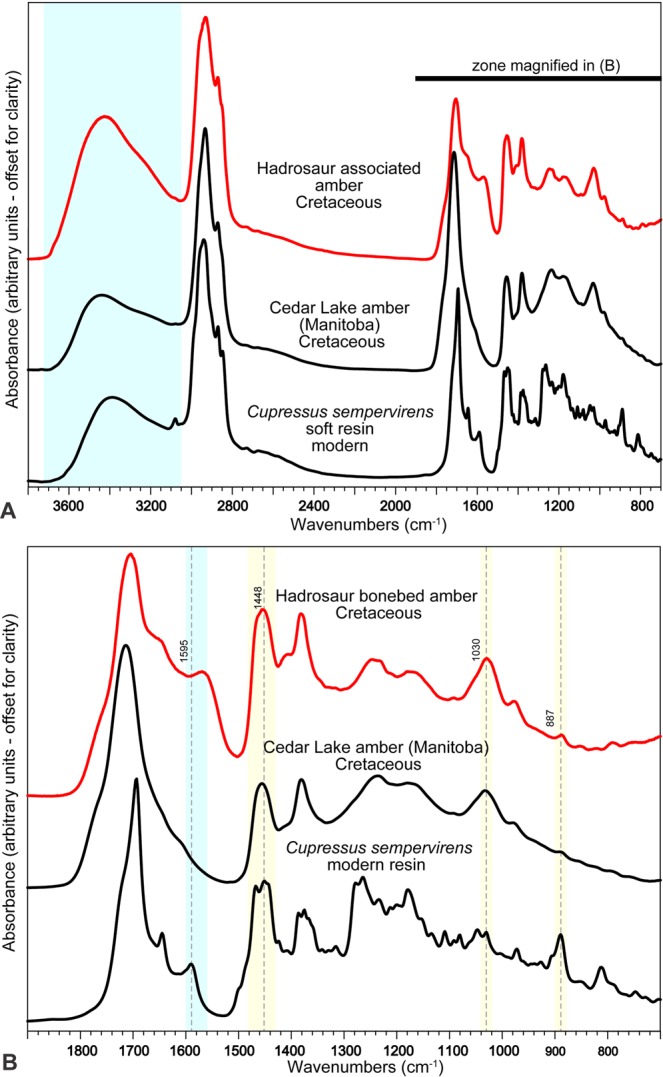
FTIR spectrum of UALVP 53367 (red line). **(A)** Comparison to oxidized cupressaceous-araucarian Cretaceous Cedar Lake amber (Manitoba), and detailed Recent Cupressaceae resin (*Cupressus sempervirens*). Hydroxyl groups region underlain in blue. **(B)** Magnified spectral region 700–1900 cm^−1^; numbers refer to spectral features mentioned in text; blue marks regions linked to molecular water, yellow marks characteristics of cupressaceous-araucarian resins.

Oxidation and dark drying lines restrict visibility to the outer 1–2 mm of the amber, but within this window, a partial aphid inclusion is visible on the convex (lingual) side (Figs. 1, 2). Numerous fragments of indeterminate plant material, and translucent structures that likely represent fungal hyphae, are also scattered throughout the amber. However, no unobscured view is available, due to internal fractures and flow lines (Figs. 1,2). At its thickest point, the carbon film along the labial surface of the amber piece is less than 0.5 millimeters thick (Fig. 3D,F,G): it is thin and sporadic in occurrence, appearing to preserve fragments of foliage, as opposed to a larger wood fragment.

### Palaeoentomology

The insect inclusion (Fig. 2) clearly belongs to Aphidoidea, with at least one conical siphunculus visible, and no ovipositor^7^. It meets all diagnostic criteria for Cretamyzidae, a monotypic family of extinct aphids previously documented from the slightly older amber of the Foremost Formation at Grassy Lake, Alberta^7,8^. Aside from its broader anterior body shape, and lack of a pointed frons (a region not preserved), the new specimen is indistinguishable from *Cretamyzus pikei* Heie, 1992^7^. Most notably, it shares a unique, elongate, antennal scape with a bulbous base; laterally projecting eyes; elongate, narrow rostrum; and six-articled antennae. Furthermore, UALVP 53367 has peculiar lateral ‘hairs’ known from the abdominal segments of *C. pikei*^7^, but these appear to be fewer and with broader bases. Considering the shapes and preservation of these projections, their identification as waxy secretions or setae^7^ seems less likely than weakly sclerotized cuticle. A small, rounded cauda and anal plate are clearly visible at the tip of abdomen, dorsal to an exposed aedeagus with parameres. Comparable genitalia have been suggested for other fossils^9^ and observed in modern aphids^e.g.,^^10^. Based on elongate claws and mouthparts, Cretamyzidae have been postulated to feed in bark crevices on resin-producing trees^7^. Shared morphology and entrapment within a multi-flow piece of amber support a similar habitat for the UALVP 53367 aphid.

### Amber characterization

The infrared spectrum of UALVP 53367 amber was analyzed to assess its source tree. The specimen is typical for terpenoid-based resins of the period, yet there is an intense and broad absorption peak between 3100 and 3700 cm^−1^, attributable to the presence of hydroxyl (OH) groups^11^. Additionally, the spectral range below 1800 cm^−1^ is characterized by unusually smooth absorption peaks lacking small-scale features, similar to partially oxidized ambers with comparable reddish coloration (Fig. 3). The smoothness of spectral features indicates loss of detail during the partial breakdown of the original macromolecular structure of the resin’s terpenoid constituents. However, enough diagnostic features remain to permit classification as a cupressaceous-araucarian^12^ or Class Ib^13^ resin. Diagnostic spectral features include absorption peaks at 1448, 1030, and 887 cm^−1^. Further differentiation between the conifer families Araucariaceae and Cupressaceae is usually not possible, due to their similar terpene profiles^13^. The absorption peak between 1560 and 1600 cm^−1^ is most likely related to the presence of molecular water, which has its H-O-H bending mode^14^ located at 1595 cm^−1^. The presence of molecular water, as opposed to structurally bound hydroxyl groups, is uncommon in fossil resins, but occurs in some modern cupressoid taxa (*e.g*., *Cupressus sempervirens*, Mediterranean cypress, Fig. 3). Because the amber fragments analyzed did not contain any visible inclusions, molecular water is most likely dispersed within terpene skeletal structures.

The occurrence of cupressaceous-araucarian resins within the Dinosaur Park Formation is not unusual, as most Canadian Cretaceous ambers analyzed to date are of this type^8,12,15^. Many Late Cretaceous ambers from the region also have inclusions of cupressaceous wood or foliage of *Parataxodium*^3^, suggesting that this group is responsible for most resin production in the region. Previous studies utilizing Nuclear Magnetic Resonance spectroscopy have suggested that Araucariaceae may be responsible for most Cretaceous amber deposits^15^, but NMR faces a similar difficulty to that of FTIR in resolving these two source-plant families^16^. Considering that Araucariaceae have not been recovered as inclusions, their pollen is unknown in Dinosaur Provincial Park, and their Cretaceous range does not encompass the study area^17^, a cupressaceous source tree seems more certain. This group of trees would have dominated the landscape surrounding the hadrosaur.

**Figure 4:**
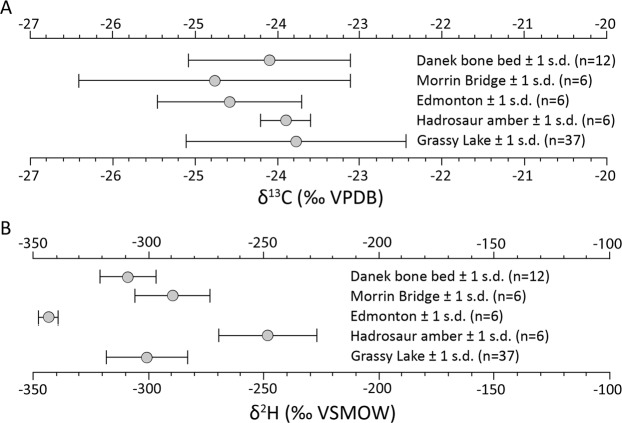
Stable isotope comparison between UALVP and surrounding ambers. (**A**) δ^13^C. (**B**) δ^2^H. Comparison to Danek Bonebed, Morrin Bridge, and Edmonton (Horseshoe Canyon Formation), and Grassy Lake (Foremost Formation) ambers, Alberta^data from^ ^1,8^.

Stable isotope analyses conducted on the amber provide proxies for various environmental conditions during resin production. UALVP 53367 yielded δ^13^C values ranging from −24.2 to −23.6‰, (mean −23.9 ± 0.28‰, *n* = 6), and δ^2^H values ranging from −268.9 to −227.0‰ (mean −248.4‰ ± 14.05‰, *n* = 6; Fig. 4, Table S1). These values fall within the known range for amber, and variability between samples drawn from the amber piece can be attributed to plant particulate inclusions. These were unavoidable in sampling and are isotopically heavier in both C and H^18,19^. Values from UALVP 53367 are similar to those obtained in regional analyses of amber from the Foremost Formation, Taber Coal Zone (which underlies the Oldman Formation, beneath the Dinosaur Park Formation—mean δ^13^C value −23.7‰, mean δ^2^H value 297.8‰), as well as the Horseshoe Canyon Formation. (which overlies the marine Bearpaw Formation, above the Dinosaur Park Formation—mean δ^13^C value −24.1‰, mean δ^2^H value −308.4‰)^4,8^.

## Discussion

UALVP 53367 has δ^13^C values similar to adjacent deposits, matching well with large-scale secular trends in amber δ^13^C values^4^. This consistency suggests resin production by healthy plants experiencing little or no anomalous physiological stress^20,21^. Instead, the large, multilayered resin flow is more consistent with repair following physical injury to the tree. If it is representative of general resin production at the time, it would reflect an atmospheric partial pressure of oxygen of roughly 13–14%, as opposed to the modern 21%^4^. The relationship between amber and bulk plant δ^13^C values^4,21^ also establishes a value of approximately −23.9‰ C3 plants at the time—providing a baseline for food web analysis and partially explaining the elevated values observed in Late Cretaceous hadrosaur teeth^e.g.,^^22,23^. Ultimately, such inferences will remain speculative until more data are gathered from surrounding strata, and the relationships between resin compositions, atmospheric oxygen, and carbon dioxide are better understood.

Despite similarity in δ^13^C values, δ^2^H values in UALVP 53367 are approximately 50–60‰ higher and internally variable compared to nearby deposits. This suggests that UALVP 53367 was formed under a different hydrological regime, and that multiple resin flows may record hydrological variations or variable particulate content. A relatively consistent fractionation factor of approximately −200 to −230‰ has been observed between local meteoric water and the resin produced by C3 plants accessing this water^18,19,24,25^. If applied to UALVP 53367, the inferred mean δ^2^H value for local waters would be approximately −48 to −18‰, as opposed to the continental −134.6 to −130.5‰ observed in southern Alberta mean annual precipitation now^26^. This fits well with paleogeographic and paleoenvironmental reconstructions of the upper Dinosaur Provincial Park Formation as an alluvial-coastal plain undergoing transgression^6,23^. The observed deuterium enrichment indicates that water accessed by the amber-producing forest was advected primarily from the Western Interior Seaway, without significant transport inland or upslope. If it is assumed that the position of the excavation relative to the global meteoric water line has not changed significantly since the Cretaceous, the observed δ^2^H values suggest summer resin production at an instantaneous temperature of ≥33 °C (Fig. S1).

Judging from the impression of the dental battery on the amber surface (Fig. 1), the resin was relatively fresh and pliable when association with the dentary occurred. A thin layer of sediment between the amber and dentary suggests contact due to fluvial transport, not interactions during life. It was initially hoped that the amber surface might contain traces of soft tissue or decay products from the hadrosaur. However, the thin carbon film preserved adjacent to the dentary did not yield elevated δ^13^C values (Table S1), suggesting a source related to the resin-producing tree, not the corpse. Because the dentary was recovered as a relatively unweathered, disarticulated element missing its teeth, the amber must have clung to it after decay had removed the flesh, but before the bone had undergone significant transport.

The series of events required for preservation of insect-bearing amber in direct association with dinosaur remains is remarkable. As was suggested in the original work on Cretamyzidae^7^, the aphid’s presence within a multiple-flow amber piece suggests entrapment while feeding on the resin-producing tree. The resin mass then entered a nearby river system at approximately the same time as skeletal material from a hadrosaur, and was pressed onto the bone as a result of transport after the body had disarticulated and decayed. Subsequent disturbances flattened the resin mass, but its association with the bone was maintained throughout the burial and diagenesis processes: shifting sediments only created minor fractures in the amber after polymerization.

If the association between resin and dinosaur remains had taken place at a slightly different stage in the taphonomic process, the amber described herein might have provided microscopic details of the hadrosaur itself—like recent discoveries of coelurosaur and enantiornithine remains in Burmese amber (Myanmar, ~99 Ma)^e.g.,^^27–30^. Amber from the Foremost Formation has already yielded a diverse suite of integumentary structures^31^, and UALVP 53367 emphasizes the potential for fossiliferous ambers to provide insight into dinosaur paleoecology rather than largely representing the purview of arthropod paleontology. In addition to providing paleoecological information, future finds of bonebed amber may provide insights that are not available from skeletal remains alone, and they certainly warrant attention during the excavation and preparation processes.

## Methods

The amber sample is exceptionally friable and large compared to most of the specimens recovered from adjacent formations in the region^3^. Its exposed surface was supported with “five-minute epoxy”, in order to separate it from the dentary without crumbling. Milligram scale amber fragments from the freshly exposed surface were gathered for stable isotope and Fourier-transform Infrared spectroscopy (FTIR) analyses, then the main specimen was vacuum-injected with a low viscosity, mineralogical grade epoxy (Buehler EpoThin) for stabilization^32^. The initial support epoxy and excess material from the vacuum-injection were removed with rotary tools and wet sandpaper of varying grits, returning the specimen to approximately its original shape, and removing much of the sediment and carbon film that were obscuring the labial side. The insect inclusion was extracted using a razor saw, and slide-mounted using standard techniques^32^ to improve visibility.

Stable isotope analyses were carried out on six amber samples (both adjacent to and removed from the dentary surface), using standard techniques for offline gas extraction ^e.g.,^^4,20^. Stable isotopic compositions of carbon are expressed in delta notation relative to the Vienna Pee Dee Belemnite standard (δ13C, VPDB) for carbon, and relative to Vienna standard mean ocean water (δ2H, VSMOW) for hydrogen. The precision for the procedures outlined here are ±3‰ for δ2H and ±0.1‰ for δ13C. Gar (Lepisosteidae) teeth were also found in association with UALVP 53367, but these lacked sufficient mass for supplementary analysis: there were also no isolated hadrosaur teeth from which to draw enamel for analysis. One of the six samples analyzed was likely contaminated during preparation, repeatedly presenting δ2H values outside the known range for amber (97.3‰, 116.2‰)^19^, as well as a δ13C value outlier (−26.8‰), that have been excluded from the results. A second sample was lost to leakage during offline gas extraction.

Infrared absorption spectra of the amber were obtained to determine botanical source and assess the state of preservation. The spectra were collected using a Bruker Vertex 70 FTIR spectrometer and Hyperion 3000 IR microscope. Samples were freshly broken amber fragments with thickness ≤5 μm, to retain infrared transparency, and diameters of 50–100 μm. Spectra were collected from 700–4000 cm^−1^ with a spectral resolution of 1 cm^−1^. For each sample and background spectrum, 80 interferograms were collected and co-added. No additional treatment of the resulting spectra was performed. Final spectra were compared to an existing spectral library of modern and fossil resins using established characteristics^12^.

All specimens are available through UALVP museum collection; and correspondence should be directed to RCM (ryan.mckellar@gov.sk.ca.)

## Supplementary information

Supplementary Information File

## Data Availability

All data are available in main text or supplementary materials. The specimen studied (UALVP 53367) is deposited in the University of Alberta Laboratory for Vertebrate Palaeontology collection, Edmonton, Alberta, Canada.
