# Supplementary material for: A direct association between amber and dinosaur remains provides paleoecological insights
Source: Sci Rep. 2019 Nov 29;9:17916. doi: 10.1038/s41598-019-54400-x (PMC6884503; doi:10.1038/s41598-019-54400-x)
Supplement: Supplementary file 1 — Supplementary Information File [file 41598_2019_54400_MOESM1_ESM.pdf]

Supplementary Information for

A direct association between amber and dinosaur remains provides paleoecological insights

Ryan C. McKellar, Emma Jones, Michael S. Engel, Ralf Tappert, Alexander P. Wolfe, Karlis Muehlenbachs, Pierre Cockx, Eva B. Koppelhus, Philip J. Currie

This PDF file includes:

Supplementary Figure S1
Supplementary Table S1

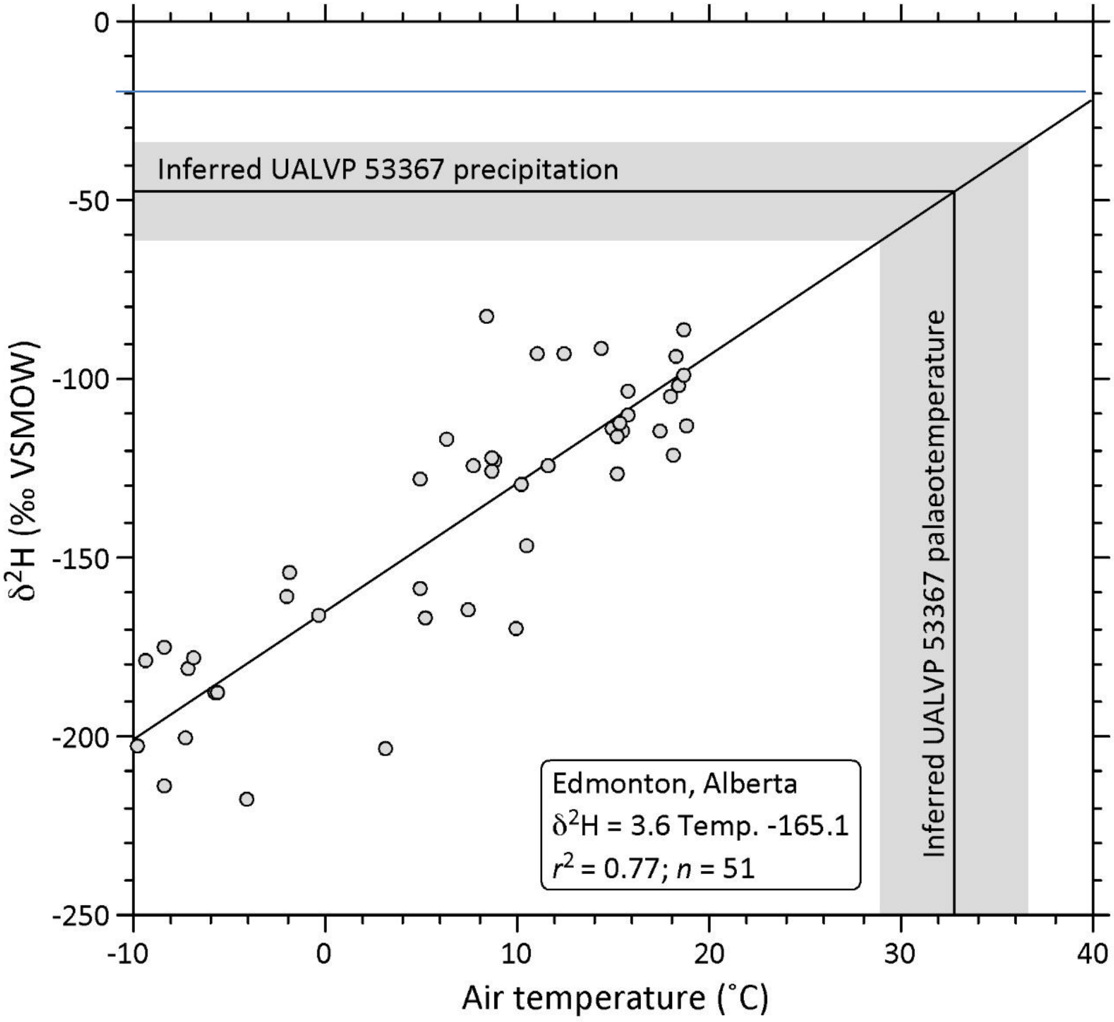

Supplementary Figure S1.

Inferred temperature during resin production, based on the modern local meteoric water line for Edmonton, Alberta, Canada. If a greater fractionation between environmental water and amber is utilized (i.e., -230‰ instead of -200‰), temperature estimates are driven to higher values, with a mean at 40°C.

Sample ID*:	δ^2H (‰ VSMOW)	δ^{13}C (‰ VPDB)	Proximity to bone	Sample mass (mg)
A1	-268.86	-24.19	free-floating	4.7
A1-2	-251.43	-23.95	free-floating	3.3
A3	-249.33	-24.22	adpressed	5.2
A5	-226.97	-23.59	adpressed	2.4
A6	-240.01	-23.58	adpressed	2.6
A6-2	-253.71	-23.89	adpressed	3.8
Mean (s.d.)	-248.4 (14.05)	-23.9 (0.28)		

Supplementary Table S1.

Stable isotope ratios from various regions of UALVP 53367. Values from other Albertan amber deposits stem from literature[1,4,8].
